# Trends and disparities in amyloidosis and cardiovascular disease mortality: a population-based retrospective study in the United States (1999–2020)

**DOI:** 10.1186/s12872-026-05510-8

**Published:** 2026-01-30

**Authors:** Faizan Ahmed, Tehmasp Rehman Mirza, Zoha Iftikhar, Haris Bin Tahir, Fenilkumar Kotadiya, Anika Goel, Haider Hussain Shah, Saman Rauf, Yusra Junaid, Talha Qadeer, Abdul Waheed, Najam Gohar

**Affiliations:** 1 Jersey Shore University Medical Center, New Jersey, USA; 2 Shalamar Medical & Dental College, Lahore, Pakistan; 3 Karachi Medical and Dental College, Karachi, Pakistan; 4 Lahore General Hospital, Lahore, Pakistan; 5 Charleston Area Medical Center, West Virginia, USA; 6 Kakatiya medical College, Telangana, India; 7 Bayhealth Hospital, Kent Campus, Dover, USA; 8 Fatima Jinnah Medical University, Lahore, Pakistan; 9 Dow University of Health and Sciences, Karachi, Pakistan; 10 Ameer-ud-Din Medical College, PGMI, Lahore, Pakistan

**Keywords:** Amyloidosis, Cardiovascular disease, Disparities, Mortality, Trends, Epidemiology, United states

## Abstract

**Background:**

Amyloidosis is increasingly recognized as a contributor to heart failure, particularly among older adults and patients with heart failure with preserved ejection fraction (HFpEF). Despite advances in diagnostic imaging and disease-modifying therapies, amyloidosis remains underdiagnosed in many settings, and population-level data examining its co-occurrence with cardiovascular disease on death certificates are limited. This study examined two decades of national mortality data to evaluate deaths co-coded with amyloidosis and cardiovascular disease (CVD) in the United States and to assess temporal trends and demographic disparities in age-adjusted mortality rates.

**Methods:**

A retrospective analysis was conducted using mortality data from the CDC WONDER database spanning 1999–2020. Age-adjusted mortality rates (AAMRs) per 1,000,000 persons were calculated, and trends were assessed using Average Annual Percentage Change (AAPC) and Annual Percent Change (APC) using Joinpoint 5.0.2.

**Results:**

Between 1999 and 2020, 26,391 amyloidosis and CVD-related deaths occurred among adults aged 25 years and older in the United States. The overall AAMR for deaths co-coded with amyloidosis and CVD increased from 4.40 in 1999 to 9.31 in 2020, with an AAPC of 3.49 (*p* < 0.001). The most pronounced increase occurred between 2018 and 2020 (APC: 13.60). Rates were higher among men than women, with both sexes showing a marked increase in the last decade. African American or Black individuals had the highest rates (11.40), followed by White (5.11) and Hispanic (3.86) individuals. Rates were highest in the Northeast region (6.71). Metropolitan areas had higher rates than non-metropolitan areas (5.73 vs. 4.76), with a more pronounced increase in metropolitan regions.

**Conclusions:**

Age-adjusted mortality rates for deaths co-coded with amyloidosis and cardiovascular disease have increased over time, likely reflecting improved recognition and documentation. Higher rates of co-coded deaths were noted among men, African Americans, and individuals in the Northeast region, highlighting potential disparities in diagnostic access and recognition.

**Supplementary Information:**

The online version contains supplementary material available at 10.1186/s12872-026-05510-8.

## Introduction

Amyloidosis, characterized by extracellular deposition of misfolded proteins, particularly transthyretin (ATTR) and immunoglobulin light chains (AL), is increasingly recognized as an important contributor to cardiovascular morbidity, especially among older adults and patients with heart failure with preserved ejection fraction (HFpEF) [1]. Although it was previously regarded as a rare condition, recent diagnostic procedures having high sensitivity and specificity such as cardiac MRI, bone scintigraphy and AI assisted imaging have led to a significant rise in its detection [2, 3]. ATTRwt (wild type form of ATTR) is generally found in men with ages above 70, whereas ATTRv (hereditary ATTR) is caused by pathogenic gene mutations in the TTR gene which might present earlier [1].

Management is improving with recent therapeutic developments. A next-generation TTR stabilizer approved in 2024, acoramidis, has reduced mortality and morbidity in ATTR cardiomyopathy (ATTR-CM), and RNA-silencing medicines such as patisiran have also shown mortality and morbidity benefits in ATTR-CM [4, 5]. These options are a shift towards disease-modifying treatment, unlike the previous palliative care approach.

Despite these advances, amyloidosis is still vastly underdiagnosed, presenting with nonspecific symptoms or symptoms resembling other cardiac conditions. It has been speculated through autopsies and clinical studies that many older adults with HFpEF have amyloid deposits [6]. When amyloidosis coexists with cardiovascular disease, it is associated with progressive clinical deterioration and adverse outcomes, particularly in the absence of timely recognition and targeted management.

Moreover, there is a racial and socioeconomic discrepancy. Black patients are affected more often with ATTRv because of the V122I mutation, and face higher in-hospital deaths, longer hospitalizations, and increased complications after being diagnosed [7]. Such disparities show systematic disparities in access to advanced diagnostics and treatment.

The population-level data on mortality due to amyloidosis and CVD is scanty. Vast majority of studies investigate each of these diseases independently, overlooking the intersectional mortality burden which may compound health inequities. This study attempts to address this gap by using 20 years of U.S. national mortality data to evaluate trends and disparities in Amyloidosis and Cardiovascular Disease (CVD)-related deaths. For clarity, this study evaluates deaths in which amyloidosis and a cardiovascular condition were co-coded in the multiple-cause fields, rather than all deaths attributable solely to amyloidosis. By calculating age-adjusted mortality rates (AAMRs) and stratifying by sex, race/ethnicity, geography, and urbanization, we provide a comprehensive, demographic-driven epidemiologic profile of this emerging public health issue.

## Methods

### Population and study setting

This population-based analysis utilized publicly available mortality data from the *Centers for Disease Control and Prevention’s Wide-Ranging Online Data for Epidemiologic Research (CDC WONDER)* database, which compiles information from U.S. death certificates [8]. We examined all deaths attributed to amyloidosis (*International Classification of Diseases*,* 10th Revision [ICD-10] code E85*) and cardiovascular diseases (*ICD-10 codes I00 to I99*) occurring between 1999 and 2020 across all 50 states and the District of Columbia [9]. For this study, we defined “amyloidosis and CVD-related deaths” as death certificates in which amyloidosis (ICD-10 E85) and at least one cardiovascular disease code **(**ICD-10 I00–I99**)** were both recorded in the multiple cause of death (MCD) fields. This definition captures deaths where both conditions were co-coded, representing a subset of all amyloidosis deaths rather than total amyloidosis mortality or clinically adjudicated cardiac involvement. Adults aged 25 years and older at the time of death were included in the analysis. Because CDC WONDER provides deidentified, publicly available data, institutional review board approval was not required. The study adhered to the *Strengthening the Reporting of Observational Studies in Epidemiology (STROBE)* guidelines for observational research [10]. The analytical framework was adapted from our previously published methodology on national cardiovascular mortality trends, with modifications to address amyloidosis-related outcomes [11].

### Data abstraction

The dataset extracted from death certificates included information on year of death, place of occurrence, sex, age group, race and ethnicity, geographic region, state, and urban-rural classification. Deaths were categorized according to their location, which included hospitals, private residences, hospices, nursing homes, and long-term care facilities. Urbanization categories were based on the National Center for Health Statistics Urban-Rural Classification Scheme. Urban areas comprised large metropolitan regions with populations of one million or more, as well as medium or small metropolitan areas with populations between 50,000 and 999,999. Rural areas were defined as those with populations under 50,000, consistent with the 2013 U.S. Census definitions [12]. Geographical divisions followed the U.S. Census Bureau classification, which divides the country into four main regions namely the Northeast, Midwest, South and West.

### Statistical analysis

We calculated both crude and age-adjusted mortality rates (AAMRs) per 1,000,000 individuals for each calendar year, stratified by sex, race and ethnicity, state, and urban-rural status, with corresponding 95% confidence intervals (CIs). Crude mortality rates were derived by dividing the number of amyloidosis and CVD-related deaths by the corresponding U.S. population for each year. Age-adjusted mortality rates were calculated by standardizing amyloidosis and CVD-related deaths to the United States population in 2000. Temporal trends were analyzed using the *Joinpoint Regression Program (Version 5.0.2*,* National Cancer Institute)* to estimate the annual percent change (APC) and its 95% CI in AAMRs [13]. A two-tailed p-value of less than 0.05 was considered statistically significant.

## Results

From 1999 to 2020, Amyloidosis and CVD accounted for a total of 26,391 deaths in U.S adults aged ≥ 25 years (Supplementary Table 1). The age-adjusted mortality rate (AAMR) showed an upward trend, starting 4.4 per 1,000,000 (95% CI 4.09–4.71) in 1999 and peaking at 9.31 per 1,000,000 (95% CI 8.94–9.68) in 2020 (Supplementary Table 2).

### Annual trends in AAMR

**Fig. 1:**
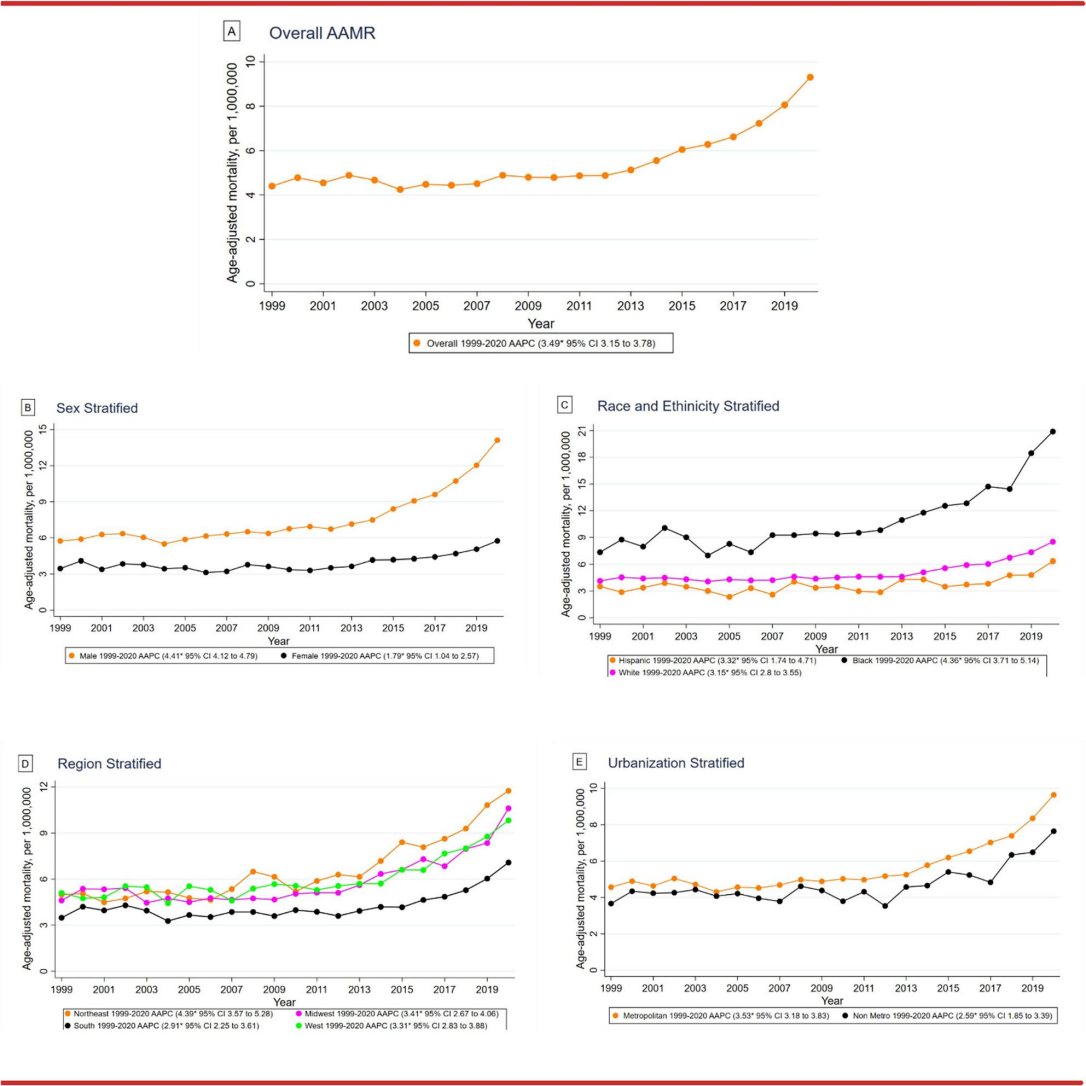
CVD-related mortality trends in Amyloidosis in the United States, 1999 to 2020 with (**A**) overall CVD-related AAMR in Amyloidosis per million, (**B**) sex-stratified CVD-related AAMR in Amyloidosis per million, (**C**) race/ethnicity Stratified CVD-related AAMR in Amyloidosis per million, (**D**) regionally stratified CVD-related AAMR in Amyloidosis per million, (**E**) urbanization stratified CVD-related AAMR in Amyloidosis per million

From 1999 to 2020, the overall AAMR for Amyloidosis and CVD-related deaths in adults inclined from 4.4 to 9.31 (AAPC: 3.49, 95% CI: 3.15 to 3.78). A slight decrease occurred between 1999 and 2012 (APC: 0.54, 95% CI: -0.37 to 1.17), followed by a moderate increase from 2012 to 2018 (APC: 6.81, 95% CI: 0.59 to 8.23) and a significant increase until 2020 (APC: 13.60, 95% CI: 8.57 to 16.77). (Fig. 1A, Supplementary Table 3).

### AAMR stratified by sex

Between 1999 and 2020, Amyloidosis and CVD accounted for 15,745 deaths in men (59.7%) and 10,646 deaths in women (40.3%). Throughout this period, men had higher AAMRs than women (Overall AAMR: Men: 7.86, 95% CI: 7.73 to 7.98; Women: 3.92, 95% CI: 3.85 to 4) (Supplementary Table 2). The overall Amyloidosis and CVD-related AAMR for both men and women increased from 1999 to 2020, with a greater decline in women (Men: AAPC: 4.41, 95% CI: 4.12 to 4.79, *p* < 0.001; Women: AAPC: 1.79, 95% CI: 1.04 to 2.57, *p* < 0.001). Specifically for men, increase occurred across the last decade [APC 2013–2018: 8.31, 95% CI: 5.37 to 9.96 (*p* < 0.001); APC 2018–2020: 15.32, 95% CI: 11.76 to 17.69 (*p* < 0.001)]. From 1999 to 2011, the AMR for women decreased (APC: -0.90, 95% CI: -4.80 to 0.66, *p* = 0.22), followed by a significant increase until 2020 (APC: 5.48, 95% CI: 3.52 to 11.25, *p* < 0.001). (Fig. 1B, Supplementary Table 3).

### AAMR stratified by race/ethnicity

Significant variability in Amyloidosis and CVD-related deaths was observed among different racial/ethnic groups from 1999 to 2020. The highest number of deaths occurred in Whites (19,321, 73.2%), followed by Black or African Americans (4873, 18.5%), Hispanic or Latinos (1386, 5.3%), Asian or Pacific Islanders (678, 2.6%), and the lowest in American Indian or Alaska Natives (79, 0.3%). Overall, the Amyloidosis and CVD-related AAMRs were highest among Black or African Americans, followed by Whites and Hispanic or Latinos. (Overall AAMR: Black or African American: 11.4, 95% CI: 11.07 to 11.73; White: 5.11, 95% CI: 5.04 to 5.18; Hispanic or Latino: 3.86, 95% CI: 3.65 to 4.07). From 1999 to 2020, Amyloidosis and CVD-related AAMRs increased for Black or African Americans (AAPC: 4.36, 95% CI: 3.71 to 5.14, *p* < 0.001), Whites (AAPC: 3.15, 95% CI: 2.80 to 3.55, *p* < 0.001), and Hispanic or Latinos (AAPC: 3.32, 95% CI: 1.74 to 4.71, *p* < 0.001). (Fig. 1C, Supplementary Tables 3 and 4).

### AAMR stratified by geographical regions

From 1999 to 2020, the distribution of Amyloidosis and CVD -related deaths across the U.S. census regions showed 6231 (23.6%) deaths in the Northeast, 6309 (23.9%) in the Midwest, 7574 (28.7%) in the South, and 6277 (23.8%) in the West. On average, the highest Amyloidosis and CVD-associated mortality rates were observed in the Northeast (AAMR: 6.71, 95% CI: 6.54 to 6.88), followed by the West (AAMR: 6.18, 95% CI: 6.03 to 6.34), the Midwest (AAMR: 5.9, 95% CI: 5.76 to 6.05), and the South (AAMR: 4.34, 95% CI: 4.24 to 4.44). Overall, the AAMR associated with Amyloidosis and CVD increased in the all the regions from 1999 to 2020 [Northeast: AAPC: 4.39, 95% CI: 3.57 to 5.28 (*p* < 0.001); Midwest: AAPC: 3.41, 95% CI: 2.67 to 4.06 (*p* < 0.001); South: AAPC: 2.91, 95% CI: 2.25 to 3.61 (*p* < 0.001); West: AAPC: 3.31, 95% CI: 2.83 to 3.88 (*p* < 0.001)]. (Fig. 1D, Supplementary Tables 3 and 5).

### AAMR stratified by urbanization

Metropolitan areas consistently had higher AAMRs associated with amyloidosis and CVD compared to the nonmetropolitan areas from 1999 to 2020, with overall AAMRs of 5.73 (95% CI: 5.66 to 5.81) and 4.76 (95% CI: 4.61 to 4.91), respectively. Both nonmetropolitan and metropolitan areas exhibited an increase in Amyloidosis and CVD-related AAMRs from 1999 to 2020 [Nonmetropolitan: AAPC: 2.59, CI: 1.85 to 3.39 (*p* < 0.001); Metropolitan: AAPC: 3.53, CI: 3.18 to 3.83 (*p* < 0.001)]. (Fig. 1E, Supplementary Tables 3 and 6).

### AAMR stratified by state

**Fig. 2:**
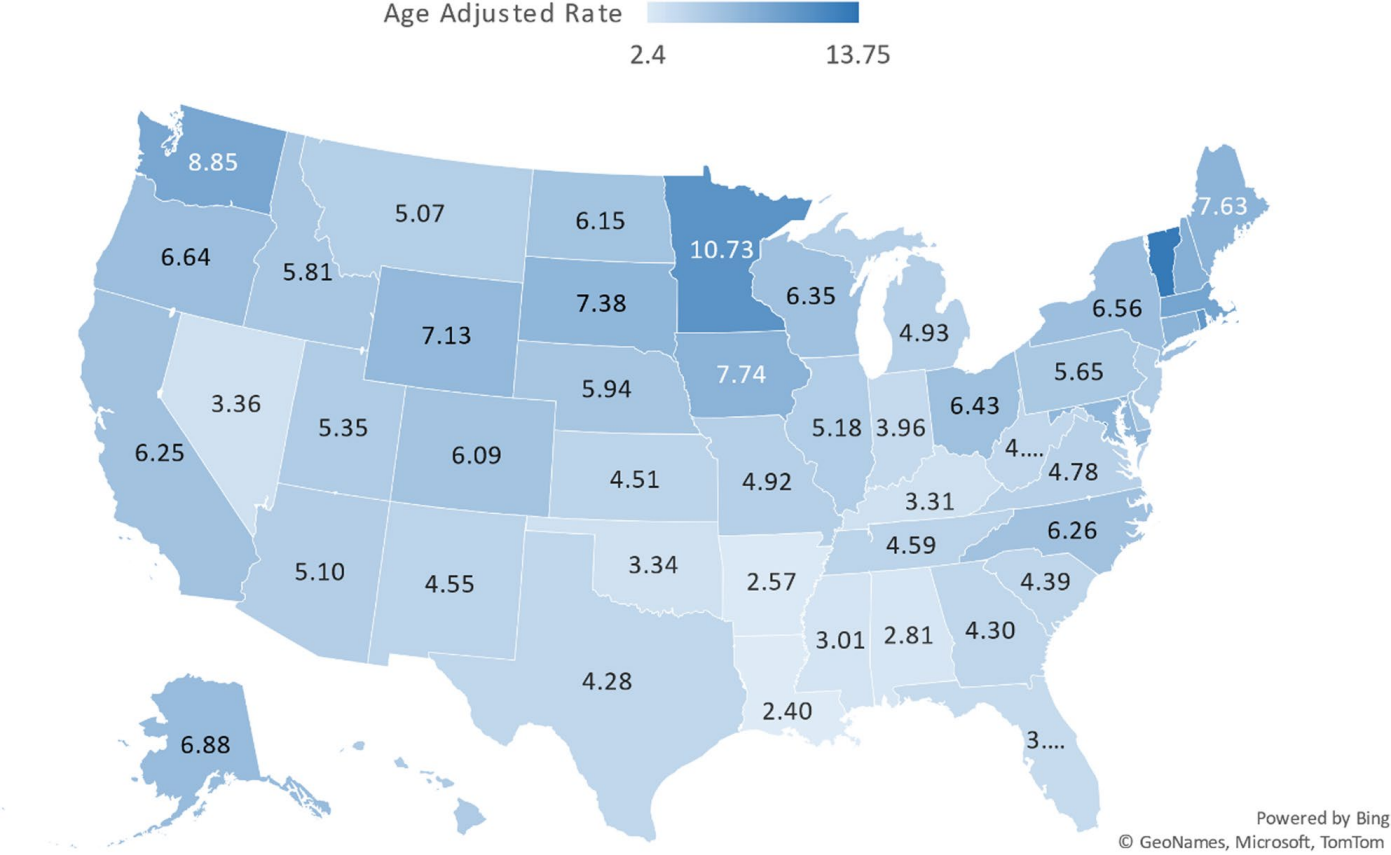
State-Stratified Amyloidosis and CVD-related AAMRs per 1,000,000 in United States 1999–2020

Amyloidosis and CVD-related AAMRs varied significantly among U.S. states from 1999 to 2020, ranging from 2.4 (95% CI: 2.03 to 2.78) in Louisiana to 13.75 (95% CI: 11.21 to 16.29) in the District of Columbia. States in the top 90th percentile for AAMRs included Massachusetts, Rhode Island, Minnesota, Vermont, and District of Columbia, which had roughly four times the AAMRs of states in the lower 10th percentile - Kentucky, Mississippi, Alabama, Arkansas, and Louisiana. (Fig. 2, Supplementary Table 7).

### AAMR stratified by place of death

**Fig. 3:**
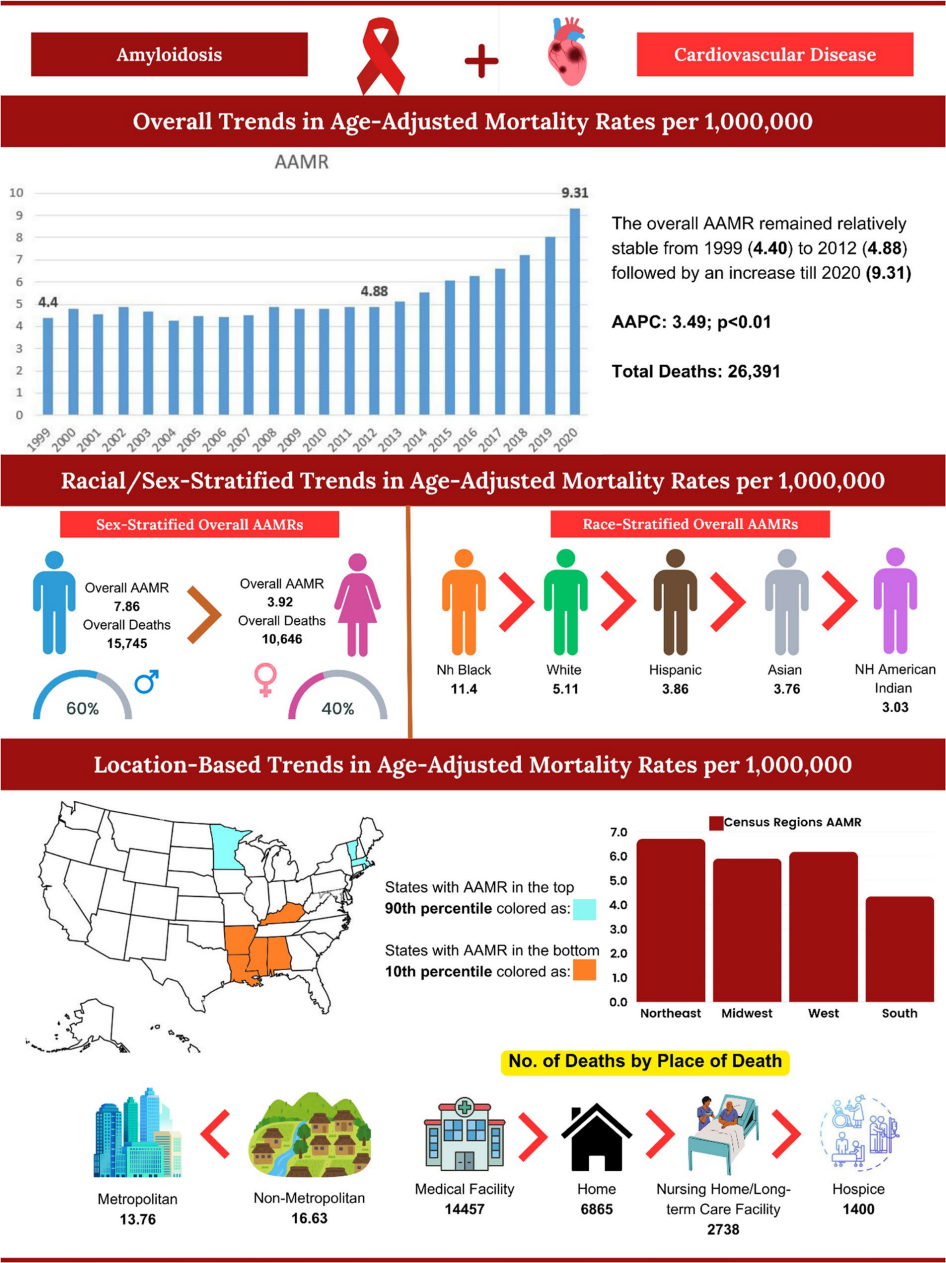
CENTRAL ILLUSTRATION: Demographic Profiles in Amyloidosis and Cardiovascular Disease-related Mortality among adults 25 to 85 + in the United States, 1999 to 2020

When stratified by the place of death, Amyloidosis-related deaths predominantly occurred in medical facilities (54.8%), followed by homes (26.0%), nursing homes/long-term care facilities (10.4%), hospice facilities (5.3%) and other/unknown locations (3.5%). (CENTRAL ILLUSTRATION [Figure 3], Supplementary Table 8)

## Discussion

The current retrospective population-based analysis describes important trends and disparities in deaths where amyloidosis and cardiovascular disease (CVD) were co-coded on U.S. death certificates from the years 1999 to 2020. The multidirectional age-adjusted mortality rates (AAMRs) reflect an emerging diagnositc and epidemiologic challenge that relates to these fatal diseases.

The increase in amyloidosis and CVD-related deaths from 4.4 per to 9.31 CVD-related deaths per 1,000,000 adults is consistent with the growing literature emphasizing the increasing clinical identification and diagnosis of amyloidosis, particularly in individuals with cardiovascular disease [14]. Current literature corroborates that increase, at least in part, with improved diagnostic experience, increased clinical identification, and more comprehensive case workup, particularly in an aging population with a high burden of cardiovascular risk factors such as hypertension, diabetes, and obesity [15].

Stratifying deaths by place of death showed that the majority of deaths were in medical venues, confirming past findings that noted the high levels of hospitalization and health care utilization of patients with amyloidosis, likely due to advanced disease manifestations and intermittent acute decompensation [16]. Furthermore, the considerable percentage of home deaths suggests missed later gap opportunities associated with diagnosis, treatment, and palliation. Future studies should build on these findings by developing studies to consider addressing these late gaps and intervention methods based in the community and in outpatient settings that could assist with detection and chronic disease management.

The sex-based stratification noted a persistent and increasing difference in rates of amyloidosis–CVD co-coded deaths between males and females, with men consistently exhibiting higher rates. This finding is similar to previous studies that have identified men as more likely to have amyloidosis identified in clinical settings and coded at the time of death. This difference may be related to biological differences, hormonal influences, and lifestyle risk factors likely influenced by the higher rates of tobacco use, and possibly higher rates of metabolic syndrome, among men [17, 18]. However, variation in diagnostic recognition, referral patterns, and access to specialized evaluation may also contribute to the observed sex-based differences. For women overall, while the rates of amyloidosis-CVD co-coded deaths were much lower, the marked increase over the most recent decade highlights changing patterns of recognition, underscoring the need for further study of sex-specific disease characteristics and diagnostic approaches [17].

There were significant differences noted in race/ethnic-based analyses, with indications that Black or African American patients had higher rates of amyloidosis-CVD co-coded deaths than Whites. This is in line with some literature that discusses racial disparities, particularly as they relate to genetic traits, social status, access of care, and possible differences regarding awareness of disease and management practices for individual races [19, 20]. Importantly, these observed differences may also reflect variation in diagnostic recognition, referral patterns, and access to specialized evaluation rather than true differences in disease-specific mortality. The increase observed among Hispanic or Latino individuals likely reflects changing patterns of recognition and documentation, potentially influenced by demographic shifts, a rising burden of cardiometabolic risk factors, and evolving healthcare utilization within these populations.

Geographically based stratification of age-adjusted rates revealed significant regional differences, with higher rates of deaths co-coded with amyloidosis and CVD in the Northeast and West compared with the Midwest and South. These differences are likely related to regional differences in healthcare capacity and access, diagnostic capabilities, and the availability of specialized evaluation and treatment for amyloidosis [21]. Differences in access to centers with advanced imaging capabilities may lead to higher detection and documentation rates in more developed urban areas. Other research has shown that there are regional differences in healthcare access and practice patterns with calls by others for use of a uniform, national guideline to ensure the same protocol and prevention is used regardless of race or geographic location of management and treatment of amyloidosis [22]. Differences in educational attainment across states may also influence healthcare-seeking behavior, contributing to higher rates of amyloidosis recognition and documentation.

In metropolitan versus nonmetropolitan areas, rates of deaths co-coded with amyloidosis and CVD were consistently higher in metropolitan areas, and both areas exhibited an increase in Amyloidosis and CVD-related AAMRs from 1999 to 2020 suggesting the potential presence of urban-rural effects stemming from differences in access to healthcare, diagnostic capabilities, specialized treatment options, and multiple other social determinants of health that may affect the ability to properly manage and outcome from disease [23, 24]. Improving rural health disparities through telemedicine, and increasing access to specialized care through implementation of decentralized care models to help mitigate observed regional disparities in documented amyloidosis–CVD deaths, may have a more significant impact than other interventions [25].

Marked state-level variation was observed in rates of deaths co-coded with amyloidosis and CVD, with far higher rates in states such as Massachusetts, Rhode Island, and the District of Columbia, compared to many southern states. These observed differences are likely a reflection of geographical diagnostic behaviors and access to specialized amyloidosis care centers being clustered in certain geographical areas [21]. This suggests that expanding diagnostic infrastructure, clinical expertise, and specialty care networks in underserved areas may reduce observed disparities in amyloidosis–CVD co-coded deaths and improve equity in disease recognition and care pathways [26].

### Limitations

There are a number of limitations in this study that need to be factored in. Most importantly, this analysis relies on death certificate data from the CDC WONDER database, which reflect coding and documentation practices rather than clinically adjudicated disease. As a result, increases in amyloidosis–CVD co-coded deaths over time may partly reflect improved diagnostic recognition and documentation rather than a true rise in disease-specific mortality. Similar diagnostic and coding challenges have been described in other chronic conditions such as diabetes mellitus, where cardiovascular complications are increasingly documented over time as clinical awareness improves [27].

First, the use of death certificate data from the CDC WONDER database can lead to misclassification bias due to underreporting or incorrect coding of amyloidosis, especially when CVD is the main clinical concern at the time of death. Secondly, the ICD-10 E85 does not show distinction between the sub-types of amyloidosis (e.g. AL and ATTR) nor does it specify the presence of cardiac involvement, which limits the granularity of the analysis. Thirdly, our outcome reflects co-occurrence of amyloidosis and CVD codes in the multiple-cause-of-death fields. ICD-10 E85 neither specifies amyloidosis subtype (AL vs. ATTR) nor confirms cardiac involvement, and therefore the results should not be interpreted as disease-specific cardiac amyloidosis mortality. Future studies should link death certificate data with clinical registries, imaging repositories, or insurance claims to verify cardiac involvement, differentiate amyloidosis subtypes, and generate more clinically actionable screening or diagnostic recommendations.

Fourth, the study uses aggregate data at the population level instead of clinical data at the patient level. This retrospective nature precludes causal inference. This does not allow adjustment for comorbidities, socioeconomic status, treatment regimens or diagnostic modalities, which influence observed mortality trends. Fifth, temporal changes in diagnostic capabilities, such as the increased use of cardiac MRI and bone scintigraphy in recent years, may have artificially inflated detection rates and associated mortality reporting in the latter part of the study period. And finally, geographical and racial variation seen in the analysis can be concealing system-wide inequity in health-care access, with underdiagnosis in underserved regions, as opposed to genuine biological differences. While age-adjusted rates mitigate some of these concerns, residual confounding cannot be ruled out.

## Conclusion

The frequency of deaths in which amyloidosis and cardiovascular disease were co-coded on U.S. death certificates has increased over the past two decades, with a more pronounced rise observed in the last decade. These patterns were disproportionately observed among men, African American communities, and individuals residing in the Northeast and metropolitan areas and likely reflect evolving diagnostic recognition, documentation practices, and access to specialized evaluation.

Geographic and demographic variation in co-coded deaths suggests potential inequities in diagnostic access, clinical suspicion, and delivery of specialized care. In light of the ongoing growth in awareness of amyloidosis, healthcare systems need to focus on an earlier diagnosis, particularly among those considered high-risk groups, and provide equal access to new diagnostic and treatment services.

These population-based data are crucial to inform resource assignment, screening and future research on disease mechanisms and disparities. To better define disease dynamics and inform clinical decision-making, future investigations should include patient-level clinical variables and subtype-specific data.

## Supplementary Information

Supplementary Material 1.

## Data Availability

All data used in this study are publicly available through the Centers for Disease Control and Prevention Wide-ranging Online Data for Epidemiologic Research (CDC WONDER) database. The authors obtained access in accordance with the CDC’s data use guidelines.
