# Supplementary material for: Trends and disparities in amyloidosis and cardiovascular disease mortality: a population-based retrospective study in the United States (1999–2020)
Source: BMC Cardiovasc Disord. 2026 Jan 30;26:184. doi: 10.1186/s12872-026-05510-8 (PMC12930566; doi:10.1186/s12872-026-05510-8)
Supplement: Supplementary file 1 — Supplementary Material 1. [file 12872_2026_5510_MOESM1_ESM.docx]

**Supplementary Data**

**Supplementary Table 1 Number of Amyloidosis and CVD-Related Deaths, Stratified by Sex and Race in Adults in the United States 1999-2020.**

| **Year** | **Overall** | **Women** | **Men** | **NH White** | **NH Black or African American** | **NH Asian or Pacific Islander** | **NH American Indian or Alaska Native** | **Hispanic or Latino** | **Population** |
| --- | --- | --- | --- | --- | --- | --- | --- | --- | --- |
| **1999** | 778 | 357 | 421 | 612 | 110 | 15 | Suppressed | 34 | 180408769 |
| **2000** | 856 | 423 | 433 | 674 | 135 | 14 | Suppressed | 28 | 181984640 |
| **2001** | 830 | 355 | 475 | 660 | 123 | 10 | Suppressed | 34 | 184305128 |
| **2002** | 906 | 410 | 496 | 680 | 163 | 17 | Suppressed | 42 | 186208028 |
| **2003** | 875 | 409 | 466 | 666 | 145 | 17 | 0 | 44 | 188090429 |
| **2004** | 812 | 372 | 440 | 634 | 120 | 18 | Suppressed | 36 | 190205384 |
| **2005** | 874 | 392 | 482 | 676 | 146 | 21 | Suppressed | 29 | 192551384 |
| **2006** | 877 | 358 | 519 | 677 | 132 | 21 | Suppressed | 44 | 195019359 |
| **2007** | 908 | 370 | 538 | 679 | 168 | 22 | Suppressed | 38 | 197403777 |
| **2008** | 1006 | 443 | 563 | 755 | 169 | 16 | Suppressed | 60 | 199795090 |
| **2009** | 1002 | 432 | 570 | 732 | 181 | 35 | Suppressed | 51 | 202107016 |
| **2010** | 1014 | 405 | 609 | 756 | 173 | 22 | Suppressed | 57 | 203891983 |
| **2011** | 1056 | 412 | 644 | 789 | 186 | 26 | Suppressed | 52 | 206592936 |
| **2012** | 1095 | 445 | 650 | 803 | 205 | 28 | Suppressed | 52 | 208826037 |
| **2013** | 1161 | 462 | 699 | 817 | 227 | 30 | Suppressed | 83 | 211085314 |
| **2014** | 1300 | 545 | 755 | 923 | 256 | 31 | Suppressed | 83 | 213809280 |
| **2015** | 1434 | 565 | 869 | 1018 | 280 | 56 | Suppressed | 74 | 216553817 |
| **2016** | 1531 | 581 | 950 | 1103 | 293 | 42 | Suppressed | 80 | 218641417 |
| **2017** | 1640 | 619 | 1021 | 1151 | 340 | 53 | Suppressed | 88 | 221447331 |
| **2018** | 1856 | 675 | 1181 | 1328 | 346 | 63 | Suppressed | 110 | 223311190 |
| **2019** | 2094 | 737 | 1357 | 1458 | 454 | 53 | Suppressed | 115 | 224981167 |
| **2020** | 2486 | 879 | 1607 | 1730 | 521 | 68 | 12 | 152 | 226635013 |
| **Total** | 26391 | 10646 | 15745 | 19321 | 4873 | 678 | 79 | 1386 | 4473854489 |

**Supplementary Table 2 Overall and Sex‐Stratified Amyloidosis and CVD–related Age-Adjusted Mortality Rates per 1,000,000 in Adults in the United States 1999-2020**

| Age-Adjusted Rate (95% CI) | | | |
| --- | --- | --- | --- |
| Year | **Men** | **Women** | **Overall** |
| **1999** | 5.73 (5.17 to 6.28) | 3.46 (3.1 to 3.82) | 4.4 (4.09 to 4.71) |
| **2000** | 5.88 (5.31 to 6.44) | 4.09 (3.7 to 4.48) | 4.78 (4.46 to 5.1) |
| **2001** | 6.27 (5.7 to 6.84) | 3.39 (3.03 to 3.74) | 4.55 (4.24 to 4.86) |
| **2002** | 6.34 (5.78 to 6.91) | 3.85 (3.48 to 4.23) | 4.89 (4.57 to 5.21) |
| **2003** | 6.03 (5.47 to 6.58) | 3.78 (3.41 to 4.15) | 4.67 (4.36 to 4.98) |
| **2004** | 5.49 (4.97 to 6.02) | 3.44 (3.09 to 3.79) | 4.25 (3.96 to 4.55) |
| **2005** | 5.86 (5.33 to 6.4) | 3.53 (3.18 to 3.88) | 4.48 (4.19 to 4.78) |
| **2006** | 6.14 (5.61 to 6.68) | 3.13 (2.81 to 3.46) | 4.44 (4.15 to 4.74) |
| **2007** | 6.31 (5.77 to 6.86) | 3.22 (2.89 to 3.55) | 4.51 (4.21 to 4.8) |
| **2008** | 6.5 (5.96 to 7.05) | 3.78 (3.43 to 4.14) | 4.89 (4.58 to 5.19) |
| **2009** | 6.36 (5.83 to 6.89) | 3.63 (3.28 to 3.97) | 4.8 (4.5 to 5.1) |
| **2010** | 6.75 (6.21 to 7.3) | 3.37 (3.04 to 3.71) | 4.79 (4.49 to 5.09) |
| **2011** | 6.93 (6.39 to 7.48) | 3.3 (2.98 to 3.62) | 4.87 (4.57 to 5.17) |
| **2012** | 6.72 (6.19 to 7.24) | 3.52 (3.19 to 3.85) | 4.88 (4.58 to 5.17) |
| **2013** | 7.14 (6.6 to 7.68) | 3.64 (3.3 to 3.98) | 5.13 (4.83 to 5.43) |
| **2014** | 7.48 (6.94 to 8.02) | 4.16 (3.8 to 4.51) | 5.55 (5.24 to 5.86) |
| **2015** | 8.4 (7.83 to 8.97) | 4.18 (3.83 to 4.53) | 6.05 (5.73 to 6.36) |
| **2016** | 9.07 (8.49 to 9.66) | 4.26 (3.9 to 4.61) | 6.28 (5.96 to 6.6) |
| **2017** | 9.6 (9 to 10.2) | 4.41 (4.06 to 4.77) | 6.62 (6.3 to 6.95) |
| **2018** | 10.72 (10.1 to 11.34) | 4.68 (4.32 to 5.03) | 7.23 (6.9 to 7.56) |
| **2019** | 12.04 (11.39 to 12.69) | 5.05 (4.68 to 5.42) | 8.06 (7.71 to 8.41) |
| **2020** | 14.12 (13.42 to 14.82) | 5.74 (5.36 to 6.13) | 9.31 (8.94 to 9.68) |
| **Total** | **7.86 (7.73 to 7.98)** | **3.92 (3.85 to 4)** | **5.56 (5.49 to 5.62)** |

**Supplementary Table 3 Annual Percent Change (APC) and Average Annual Percent Change (AAPC) of Amyloidosis and CVD–related Age-Adjusted Mortality Rates per 1,000,000 in Adults in the United States 1999-2020**

| Year Interval | APC (95% CI) | Year Interval | AAPC (95% CI) |
| --- | --- | --- | --- |
| Overall |  |  |  |
| 1999-2012 | 0.54 (-0.37 to 1.17) | 1999-2020 | 3.49* (3.15 to 3.78) |
| 2012-2018 | 6.81* (0.59 to 8.23) |  |  |
| 2018-2020 | 13.60* (8.57 to 16.77) |  |  |
| Men |  |  |  |
| 1999-2001 | 5.85 (-0.03 to 10.29) | 1999-2020 | 4.41* (4.12 to 4.79) |
| 2001-2004 | -3.05 (-4.75 to 2.69) |  |  |
| 2004-2013 | 2.27* (1.16 to 3.91) |  |  |
| 2013-2018 | 8.31* (5.37 to 9.96) |  |  |
| 2018-2020 | 15.32* (11.76 to 17.69) |  |  |
| Women |  |  |  |
| 1999-2011 | -0.9 (-4.8 to 0.66) | 1999-2020 | 1.79* (1.04 to 2.57) |
| 2011-2020 | 5.48* (3.52 to 11.25) |  |  |
| NH White |  |  |  |
| 1999-2013 | 0.52 (-0.31 to 1.2) | 1999-2020 | 3.15* (2.8 to 3.55) |
| 2013-2020 | 8.63* (7.18 to 10.86) |  |  |
| NH Black or African American |  |  |  |
| 1999-2012 | 1.31 (-0.73 to 2.79) | 1999-2020 | 4.36* (3.71 to 5.14) |
| 2012-2020 | 9.51* (7.47 to 12.89) |  |  |
| Hispanic or Latino |  |  |  |
| 1999-2017 | 1.14 (-3.14 to 2.85) | 1999-2020 | 3.32* (1.74 to 4.71) |
| 2017-2020 | 17.39* (4.49 to 34.97) |  |  |
| Census Region 1 – Northeast |  |  |  |
| 1999-2012 | 2 (-2.01 to 3.59) | 1999-2020 | 4.39* (3.57 to 5.28) |
| 2012-2020 | 8.39* (6.04 to 16.25) |  |  |
| Census Region 2 – Midwest |  |  |  |
| 1999-2009 | -1.07 (-6.02 to 2.99) | 1999-2020 | 3.41* (2.67 to 4.06) |
| 2009-2018 | 5.89 (-3.28 to 7.88) |  |  |
| 2018-2020 | 15.98* (7.09 to 21.69) |  |  |
| Census Region 3 – South |  |  |  |
| 1999-2015 | 0.29 (-0.93 to 1.31) | 1999-2020 | 2.91* (2.25 to 3.61) |
| 2015-2020 | 11.74* (7.7 to 21.67) |  |  |
| Census Region 4 – West |  |  |  |
| 1999-2014 | 1.01 (-0.07 to 1.93) | 1999-2020 | 3.31* (2.83 to 3.88) |
| 2014-2020 | 9.29* (6.95 to 13.68) |  |  |
| Metropolitan |  |  |  |
| 1999-2011 | 0.38 (-0.53 to 1.02) | 1999-2020 | 3.53* (3.18 to 3.83) |
| 2011-2018 | 6.14* (2.87 to 7.46) |  |  |
| 2018-2020 | 14.19* (8.85 to 17.42) |  |  |
| Non-Metropolitan |  |  |  |
| 1999-2012 | -0.28 (-2.53 to 1.19) | 1999-2020 | 2.59* (1.85 to 3.39) |
| 2012-2020 | 7.42* (5.07 to 12.3) |  |  |

*=indicates statistically significant value (p < 0.05)

**Supplementary Table 4 Amyloidosis and CVD-related Age-Adjusted Mortality Rates per 1,000,000, Stratified by Race in Adults in the United States 1999-2020**

| **Age-Adjusted Rate (95% CI)** | | | | | |
| --- | --- | --- | --- | --- | --- |
| **Year** | **NH White** | **NH Black or African American** | **NH American Indian or Alaska Native** | **Hispanic or Latino** | **NH Asian or Pacific Islander** |
| **1999** | 7.33 (5.95 to 8.72) | 7.33 (5.95 to 8.72) | Suppressed | 3.51 (2.39 to 4.99) | Unreliable (2.14 to 6.58) |
| **2000** | 8.75 (7.26 to 10.25) | 8.75 (7.26 to 10.25) | Suppressed | 2.87 (1.86 to 4.24) | Unreliable (1.51 to 5.09) |
| **2001** | 7.96 (6.54 to 9.38) | 7.96 (6.54 to 9.38) | Suppressed | 3.37 (2.29 to 4.78) | Unreliable (0.92 to 3.8) |
| **2002** | 10.06 (8.49 to 11.63) | 10.06 (8.49 to 11.63) | Suppressed | 3.9 (2.77 to 5.33) | Unreliable (1.85 to 5.26) |
| **2003** | 9.01 (7.52 to 10.5) | 9.01 (7.52 to 10.5) | Unreliable | 3.48 (2.49 to 4.74) | Unreliable (1.54 to 4.38) |
| **2004** | 6.96 (5.68 to 8.23) | 6.96 (5.68 to 8.23) | Suppressed | 3 (2.08 to 4.19) | Unreliable (1.74 to 4.79) |
| **2005** | 8.27 (6.9 to 9.64) | 8.27 (6.9 to 9.64) | Suppressed | 2.34 (1.54 to 3.4) | 3.28 (2.01 to 5.07) |
| **2006** | 7.33 (6.05 to 8.61) | 7.33 (6.05 to 8.61) | Suppressed | 3.32 (2.39 to 4.51) | 3.26 (1.96 to 5.1) |
| **2007** | 9.25 (7.82 to 10.69) | 9.25 (7.82 to 10.69) | Suppressed | 2.59 (1.79 to 3.61) | 3.22 (1.99 to 4.92) |
| **2008** | 9.25 (7.82 to 10.68) | 9.25 (7.82 to 10.68) | Suppressed | 4.05 (3.05 to 5.27) | Unreliable (1.33 to 3.92) |
| **2009** | 9.43 (8.01 to 10.84) | 9.43 (8.01 to 10.84) | Suppressed | 3.36 (2.47 to 4.46) | 4.37 (3.01 to 6.14) |
| **2010** | 9.37 (7.94 to 10.8) | 9.37 (7.94 to 10.8) | Suppressed | 3.48 (2.6 to 4.56) | 2.9 (1.79 to 4.43) |
| **2011** | 9.52 (8.11 to 10.92) | 9.52 (8.11 to 10.92) | Suppressed | 2.97 (2.19 to 3.94) | 3.22 (2.09 to 4.76) |
| **2012** | 9.81 (8.42 to 11.19) | 9.81 (8.42 to 11.19) | Suppressed | 2.86 (2.11 to 3.8) | 3.13 (2.06 to 4.56) |
| **2013** | 10.95 (9.49 to 12.41) | 10.95 (9.49 to 12.41) | Suppressed | 4.29 (3.39 to 5.37) | 3.3 (2.21 to 4.74) |
| **2014** | 11.78 (10.29 to 13.26) | 11.78 (10.29 to 13.26) | Suppressed | 4.3 (3.4 to 5.36) | 3.07 (2.07 to 4.39) |
| **2015** | 12.55 (11.03 to 14.06) | 12.55 (11.03 to 14.06) | Suppressed | 3.49 (2.72 to 4.42) | 5.12 (3.85 to 6.68) |
| **2016** | 12.83 (11.31 to 14.34) | 12.83 (11.31 to 14.34) | Suppressed | 3.72 (2.92 to 4.66) | 3.87 (2.78 to 5.25) |
| **2017** | 14.71 (13.1 to 16.32) | 14.71 (13.1 to 16.32) | Suppressed | 3.82 (3.04 to 4.75) | 4.57 (3.41 to 5.99) |
| **2018** | 14.44 (12.88 to 16.01) | 14.44 (12.88 to 16.01) | Suppressed | 4.77 (3.85 to 5.69) | 5.04 (3.85 to 6.47) |
| **2019** | 18.47 (16.73 to 20.21) | 18.47 (16.73 to 20.21) | Suppressed | 4.8 (3.9 to 5.71) | 4.06 (3.03 to 5.32) |
| **2020** | 20.9 (19.07 to 22.74) | 20.9 (19.07 to 22.74) | Unreliable (3.38 to 12.11) | 6.34 (5.31 to 7.37) | 4.97 (3.85 to 6.33) |
| **Total** | **11.4 (11.07 to 11.73)** | **11.4 (11.07 to 11.73)** | **3.03 (2.37 to 3.82)** | **3.86 (3.65 to 4.07)** | **3.76 (3.47 to 4.05)** |

**Supplementary Table 5 AMYLOIDOSIS and CVD-related Age-Adjusted Mortality Rate per 1,000,000 Stratified by Census Region in Adults in the United States 1999-2020**

| Age-Adjusted Rate (95% CI) | | | | |
| --- | --- | --- | --- | --- |
| Year | **Northeast** | **Midwest** | **South** | **West** |
| **1999** | 4.99 (4.27 to 5.71) | 4.61 (3.96 to 5.26) | 3.48 (3.02 to 3.94) | 5.1 (4.36 to 5.85) |
| **2000** | 5.06 (4.34 to 5.77) | 5.37 (4.67 to 6.07) | 4.2 (3.69 to 4.7) | 4.75 (4.04 to 5.46) |
| **2001** | 4.49 (3.81 to 5.17) | 5.34 (4.64 to 6.03) | 3.96 (3.48 to 4.45) | 4.81 (4.1 to 5.52) |
| **2002** | 4.74 (4.05 to 5.44) | 5.41 (4.72 to 6.11) | 4.29 (3.79 to 4.79) | 5.54 (4.79 to 6.29) |
| **2003** | 5.19 (4.47 to 5.9) | 4.46 (3.83 to 5.08) | 3.95 (3.47 to 4.42) | 5.47 (4.73 to 6.2) |
| **2004** | 5.15 (4.44 to 5.86) | 4.75 (4.11 to 5.4) | 3.27 (2.85 to 3.7) | 4.43 (3.78 to 5.09) |
| **2005** | 4.77 (4.08 to 5.45) | 4.5 (3.88 to 5.13) | 3.66 (3.21 to 4.1) | 5.54 (4.82 to 6.27) |
| **2006** | 4.64 (3.97 to 5.32) | 4.76 (4.12 to 5.39) | 3.53 (3.09 to 3.96) | 5.3 (4.6 to 6) |
| **2007** | 5.35 (4.63 to 6.06) | 4.66 (4.03 to 5.29) | 3.86 (3.41 to 4.32) | 4.6 (3.95 to 5.25) |
| **2008** | 6.49 (5.7 to 7.29) | 4.74 (4.12 to 5.37) | 3.86 (3.41 to 4.31) | 5.38 (4.68 to 6.08) |
| **2009** | 6.15 (5.39 to 6.92) | 4.67 (4.05 to 5.29) | 3.59 (3.17 to 4.02) | 5.67 (4.97 to 6.37) |
| **2010** | 5.19 (4.5 to 5.89) | 5.04 (4.4 to 5.68) | 3.98 (3.54 to 4.43) | 5.56 (4.87 to 6.26) |
| **2011** | 5.88 (5.14 to 6.63) | 5.12 (4.48 to 5.77) | 3.87 (3.44 to 4.3) | 5.29 (4.63 to 5.96) |
| **2012** | 6.29 (5.53 to 7.05) | 5.11 (4.48 to 5.74) | 3.6 (3.19 to 4.01) | 5.55 (4.88 to 6.22) |
| **2013** | 6.15 (5.4 to 6.9) | 5.61 (4.95 to 6.27) | 3.93 (3.51 to 4.36) | 5.7 (5.02 to 6.37) |
| **2014** | 7.18 (6.38 to 7.97) | 6.34 (5.64 to 7.03) | 4.19 (3.75 to 4.62) | 5.71 (5.04 to 6.37) |
| **2015** | 8.4 (7.53 to 9.27) | 6.61 (5.9 to 7.32) | 4.17 (3.74 to 4.59) | 6.61 (5.9 to 7.31) |
| **2016** | 8.08 (7.25 to 8.91) | 7.3 (6.56 to 8.04) | 4.64 (4.19 to 5.1) | 6.59 (5.89 to 7.28) |
| **2017** | 8.63 (7.77 to 9.49) | 6.84 (6.13 to 7.55) | 4.85 (4.39 to 5.3) | 7.67 (6.93 to 8.41) |
| **2018** | 9.29 (8.42 to 10.17) | 7.97 (7.22 to 8.73) | 5.28 (4.82 to 5.74) | 8.01 (7.26 to 8.75) |
| **2019** | 10.82 (9.88 to 11.76) | 8.35 (7.57 to 9.12) | 6.03 (5.54 to 6.52) | 8.77 (8 to 9.54) |
| **2020** | 11.75 (10.78-12.72) | 10.61 (9.75 to 11.46) | 7.08 (6.55 to 7.6) | 9.83 (9.03 to 10.64) |
| **Total** | **6.71 (6.54 to 6.88)** | **12.73 (12.52 - 12.94)** | **16.05 (15.86 - 16.24)** | **15.07 (14.83 - 15.3)** |

**Supplementary Table 6 Amyloidosis and CVD related Age-Adjusted Mortality Rates per 1,000,000 in the Metropolitan and Non-metropolitan areas in Adults in the United States 1999-2020**

|  | Age-Adjusted Rate (95% CI) | |
| --- | --- | --- |
| Year | **Metropolitan** | **Nonmetropolitan** |
| **1999** | 4.56 (4.21 to 4.9) | 3.66 (3.01 to 4.31) |
| **2000** | 4.89 (4.53 to 5.25) | 4.34 (3.63 to 5.05) |
| **2001** | 4.63 (4.29 to 4.98) | 4.23 (3.54 to 4.92) |
| **2002** | 5.04 (4.68 to 5.4) | 4.26 (3.56 to 4.95) |
| **2003** | 4.71 (4.37 to 5.05) | 4.43 (3.73 to 5.13) |
| **2004** | 4.3 (3.98 to 4.63) | 4.07 (3.4 to 4.75) |
| **2005** | 4.56 (4.22 to 4.89) | 4.21 (3.53 to 4.89) |
| **2006** | 4.52 (4.2 to 4.85) | 3.95 (3.29 to 4.6) |
| **2007** | 4.68 (4.35 to 5.01) | 3.78 (3.15 to 4.41) |
| **2008** | 4.97 (4.63 to 5.31) | 4.61 (3.91 to 5.3) |
| **2009** | 4.88 (4.55 to 5.22) | 4.38 (3.7 to 5.07) |
| **2010** | 5.02 (4.69 to 5.36) | 3.79 (3.16 to 4.42) |
| **2011** | 4.97 (4.64 to 5.3) | 4.31 (3.64 to 4.97) |
| **2012** | 5.17 (4.84 to 5.5) | 3.53 (2.94 to 4.12) |
| **2013** | 5.25 (4.92 to 5.58) | 4.57 (3.89 to 5.24) |
| **2014** | 5.77 (5.43 to 6.12) | 4.65 (3.97 to 5.32) |
| **2015** | 6.19 (5.84 to 6.55) | 5.4 (4.68 to 6.13) |
| **2016** | 6.54 (6.18 to 6.89) | 5.23 (4.51 to 5.94) |
| **2017** | 7.02 (6.65 to 7.39) | 4.83 (4.15 to 5.5) |
| **2018** | 7.39 (7.03 to 7.76) | 6.34 (5.58 to 7.11) |
| **2019** | 8.35 (7.96 to 8.74) | 6.48 (5.71 to 7.25) |
| **2020** | 9.64 (9.23 to 10.05) | 7.64 (6.82 to 8.47) |
| **Total** | 5.73 (5.66 to 5.81) | 4.76 (4.61 to 4.91) |

**Supplementary Table 7 Amyloidosis and CVD–related Age-Adjusted Mortality Rates per 1,000,000, Stratified by States in Adults in the United States 1999-2020 ranked according to Percentiles.**

| **State** | **Age Adjusted Rate** | **Percentile** | **Rank** |
| --- | --- | --- | --- |
| District of Columbia | 13.75 | 98 | 1 |
| Vermont | 13.23 | 96 | 2 |
| Minnesota | 10.73 | 94 | 3 |
| Rhode Island | 10.3 | 92 | 4 |
| Massachusetts | 9.13 | 90 | 5 |
| Washington | 8.85 | 88 | 6 |
| New Hampshire | 8.07 | 86 | 7 |
| Iowa | 7.74 | 84 | 8 |
| Connecticut | 7.65 | 82 | 9 |
| Maine | 7.63 | 80 | 10 |
| South Dakota | 7.38 | 78 | 11 |
| Maryland | 7.25 | 76 | 12 |
| Wyoming | 7.13 | 75 | 13 |
| Alaska | 6.88 | 73 | 14 |
| Oregon | 6.64 | 71 | 15 |
| New York | 6.56 | 69 | 16 |
| Ohio | 6.43 | 67 | 17 |
| Wisconsin | 6.35 | 65 | 18 |
| North Carolina | 6.26 | 63 | 19 |
| California | 6.25 | 61 | 20 |
| North Dakota | 6.15 | 59 | 21 |
| Colorado | 6.09 | 57 | 22 |
| Nebraska | 5.94 | 55 | 23 |
| Delaware | 5.91 | 53 | 24 |
| Idaho | 5.81 | 51 | 25 |
| Pennsylvania | 5.65 | 50 | 26 |
| Hawaii | 5.42 | 48 | 27 |
| Utah | 5.35 | 46 | 28 |
| New Jersey | 5.21 | 44 | 29 |
| Illinois | 5.18 | 42 | 30 |
| Arizona | 5.1 | 40 | 31 |
| Montana | 5.07 | 38 | 32 |
| Michigan | 4.93 | 36 | 33 |
| Missouri | 4.92 | 34 | 34 |
| Virginia | 4.78 | 32 | 35 |
| Tennessee | 4.59 | 30 | 36 |
| New Mexico | 4.55 | 28 | 37 |
| Kansas | 4.51 | 26 | 38 |
| South Carolina | 4.39 | 25 | 39 |
| Georgia | 4.3 | 23 | 40 |
| Texas | 4.28 | 21 | 41 |
| West Virginia | 4.26 | 19 | 42 |
| Indiana | 3.96 | 17 | 43 |
| Florida | 3.92 | 15 | 44 |
| Nevada | 3.36 | 13 | 45 |
| Oklahoma | 3.34 | 11 | 46 |
| Kentucky | 3.31 | 9 | 47 |
| Mississippi | 3.01 | 7 | 48 |
| Alabama | 2.81 | 5 | 49 |
| Arkansas | 2.57 | 3 | 50 |
| Louisiana | 2.4 | 1 | 51 |

**Supplementary Table 8 Amyloidosis and CVD-related Mortality, Stratified by Place of Death in Adults in the United States 1999-2020**

| **c** | **Deaths** | | | | |
| --- | --- | --- | --- | --- | --- |
| **Year** | **Medical Facility** | **Nursing Home/Long-term Care Facility** | **Hospices** | **Home** | **Other/Unknown** |
| **1999** | 515 | 94 | Missing | 143 | 20 |
| **2000** | 575 | 96 | Missing | 157 | 19 |
| **2001** | 530 | 102 | Missing | 158 | 30 |
| **2002** | 577 | 106 | Missing | 184 | 32 |
| **2003** | 555 | 101 | Suppressed | 184 | 20 |
| **2004** | 502 | 102 | Suppressed | 178 | 23 |
| **2005** | 574 | 71 | 14 | 188 | 22 |
| **2006** | 544 | 89 | 16 | 184 | 39 |
| **2007** | 548 | 92 | 27 | 201 | 30 |
| **2008** | 613 | 99 | 29 | 203 | 58 |
| **2009** | 567 | 92 | 33 | 246 | 59 |
| **2010** | 582 | 109 | 39 | 247 | 35 |
| **2011** | 580 | 117 | 62 | 264 | 30 |
| **2012** | 582 | 122 | 72 | 287 | 30 |
| **2013** | 643 | 104 | 65 | 292 | 53 |
| **2014** | 672 | 133 | 88 | 364 | 38 |
| **2015** | 760 | 135 | 118 | 375 | 43 |
| **2016** | 747 | 157 | 131 | 450 | 42 |
| **2017** | 789 | 169 | 141 | 481 | 56 |
| **2018** | 884 | 173 | 171 | 559 | 68 |
| **2019** | 956 | 250 | 187 | 617 | 79 |
| **2020** | 1062 | 225 | 203 | 903 | 87 |
| **Total** | 14457 | 2738 | 1400 | 6865 | 931 |
